# Compression of a Deep Competitive Network Based on Mutual Information for Underwater Acoustic Targets Recognition

**DOI:** 10.3390/e20040243

**Published:** 2018-04-02

**Authors:** Sheng Shen, Honghui Yang, Meiping Sheng

**Affiliations:** School of Marine Science and Technology, Northwestern Polytechnical University, Xi’an 710072, China

**Keywords:** underwater acoustic, ship radiated noise, mutual information, machine learning, deep learning, competitive learning

## Abstract

The accuracy of underwater acoustic targets recognition via limited ship radiated noise can be improved by a deep neural network trained with a large number of unlabeled samples. However, redundant features learned by deep neural network have negative effects on recognition accuracy and efficiency. A compressed deep competitive network is proposed to learn and extract features from ship radiated noise. The core idea of the algorithm includes: (1) Competitive learning: By integrating competitive learning into the restricted Boltzmann machine learning algorithm, the hidden units could share the weights in each predefined group; (2) Network pruning: The pruning based on mutual information is deployed to remove the redundant parameters and further compress the network. Experiments based on real ship radiated noise show that the network can increase recognition accuracy with fewer informative features. The compressed deep competitive network can achieve a classification accuracy of 89.1%, which is 5.3% higher than deep competitive network and 13.1% higher than the state-of-the-art signal processing feature extraction methods.

## 1. Introduction

Underwater acoustic targets recognition based on ship radiated noise is one of the main functions of passive sonar system. The acquired underwater acoustic signals are usually noisy due to the complexity of sound propagation in shallow sea and the frequent presence of high background noise in the sensor. Nowadays, underwater acoustic targets recognition still depends on the decision of well-trained sonarmen, but it is difficult to implement continuous monitoring and recognition. Hence, an unattended underwater acoustic targets recognition system with high recognition accuracy and efficiency needs to be developed to achieve real-time targets recognition.

In order to build an automatic underwater acoustic targets recognition system, various signal processing strategies were applied to extract features and design classifiers. In addition, feature selection and compression methods were studied to improve the classification accuracy and efficiency. The extracted features mainly include: cepstral features, waveform features, auditory features and wavelet features. Das [1] extracted spectral features and cepstral coefficients. Mel-frequency cepstral coefficients (MFCC) were applied to describe underwater acoustic targets by Zhang [2]. A vessel classifier was developed based on cepstral coefficients and Gaussian mixture models by Santos [3]. Zero-crossing features and peak-to-peak amplitude features were presented to describe rotation of propeller by Meng [4,5]. However, their performances were greatly reduced in noisy shallow sea. Azimi-Sadjadi [6] studied wavelet packets. However, it was difficult to determine the decomposition series of a wavelet for lack of prior knowledge. Siddagangaiah [7] studied the multiscale entropy method to detect and recognize underwater acoustic targets. A common shortcoming of the feature extraction methods discussed above is that they require priori training samples to perform the analysis. In the field of underwater acoustic targets classifier design, multiple support vector machine (SVM) classifiers were integrated to improve classification accuracy and robustness by Yang [8]. A neural classifier based on feed forward neural network was studied by Filho [9]. However, the convergence efficiency remains to be discussed. In the field of feature selection and compression, Yang [8] proposed feature selection algorithms for underwater acoustic target recognition. Wei [10] extracted high-order statistics features and compressed it with principal components analysis. However, the compressibility and efficiency improvement is still worth discussing.

In recent years, deep learning has made great achievements in the field of speech recognition and image recognition [11]. In the field of underwater acoustic targets recognition, Kamal [12] used deep belief network (DBN) and Cao [13] used sparse deep auto encoder. However, the deep neural networks have not improved in accordance with underwater acoustic targets recognition. Yang [14] proved that unsupervised pre-training of DBN with large number of unlabeled ship radiated noise can be viewed as regularization strategy which can help to initialize the networks. However, it did not enhance the clustering characteristics of hidden units. Shen [15] proposed an improved DBN in which the relevance of hidden units can be reduced with the help of grouping strategy and group sparse punitive function. However, it is inefficient to calculate the mutual information between any two features, especially in the case of too many hidden units. In order to compress the deep neural network, Han [16] proposed a compression strategy using pruning, trained quantization and Huffman coding. However, the network pruning according to a threshold value of connection weights failed to preserve the features with more classification information.

A compressed deep competitive network (DCN) based on competitive learning and network pruning is proposed to learn and extract features from ship radiated noise. Deep neural network integrated with competitive learning can make the samples in the same category more aggregated. The pruning based on mutual information is deployed to remove the redundant parameters and further compress the network. For lacking labeled ship radiated noise, unsupervised pre-training with a large amount of unlabeled data is introduced for initializing the networks parameters.

This paper is organized as follows. Relevant issues about the compression of deep competitive network is discussed in Section 2. Section 3 shows the experiment results and relevant discussions, followed by the conclusions in Section 4.

## 2. Compression of Deep Competitive Network

**Figure 1:**
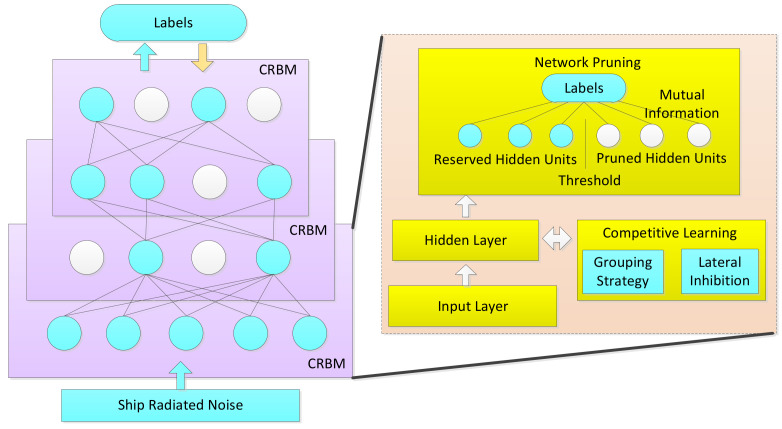
Compressed deep competitive network. The left part of the diagram shows the stacked compressed CRBM. White circles indicate the pruned hidden units. Then, the entire network is fine-tuned with class labels shown in the top of the diagram. The right part of the diagram represents the algorithm flow of compressed CRBM, where the competitive learning and the mutual information based network pruning are two core ideas of the model.

Framework of the compression of DCN is shown in Figure 1. Each layer of the network is an improved restricted Boltzmann machine (RBM) [17] integrated with competitive learning and network pruning. The training procedure is described as follows. First, RBM is pre-trained with a large amount of unlabeled data in an unsupervised learning way. Second, competitive layer is constructed by adding lateral connection among the grouped hidden units. Gradient algorithm is applied to update parameters of RBM to build the competitive restricted Boltzmann machine (CRBM). Third, mutual information is deployed to compress the CRBM by pruning the redundant hidden units and connection weights. Finally, compressed DCN is constructed by fitting a stack of compressed CRBM and the output of previous layer is input of next layer. The whole model is then discriminatively fine-tuned by a back propagation algorithm with the target of class labels.

### 2.1. Restricted Boltzmann Machine

Gaussian-Bernoulli RBM (GB-RBM) is used to deal with real-valued ship radiated noise. The visible units $v={\left(v_{1},v_{2},\ldots ,v_{n}\right)}^{T}$ are connected to hidden units $h={\left(h_{1},h_{2},\ldots ,h_{m}\right)}^{T}$ by connection weights. The connection weights and biases define a probability distribution over the joint states of the visible and hidden units via the energy function [11,17]:

(1) $$E\left(v,h|\theta \right)=\sum_{i=1}^{n}\frac{{\left(v_{i}-a_{i}\right)}^{2}}{2}-\sum_{j=1}^{m}b_{j}h_{j}-\sum_{i=1}^{n}\sum_{j=1}^{m}v_{i}W_{ij}h_{j}$$

$\theta =(W_{ij},a_{i},b_{j})$ , where $W_{ij}$ represents the connection weight between visible unit *i* and hidden unit *j*, $a_{i}$ and $b_{j}$ are biases terms. *n* and *m* are the numbers of visible and hidden units, respectively.

The conditional distribution P(h|v,θ) is $P\left(h_{j}=1|v,\theta \right)=\sigma \left(b_{j}+\sum_{i}v_{i}W_{ij}\right)$, where $\sigma \left(x\right)=1/\left(1+e^{-x}\right)$. The conditional distribution P(v|h,θ) is $P\left(v_{i}=1|h,\theta \right)=N\left(a_{j}+\sum_{j}h_{j}W_{ij},1\right)$, where N(μ,V) obeys the Gaussian distribution with mean μ and variance *V*. The parameters of RBM are optimized by the gradient:

(2) $$\frac{\partial lnp(v)}{\partial \theta }=E_{h|v}\left[\frac{\partial E(v,h)}{\partial \theta }\right]-E_{h,v}\left[\frac{\partial E(v,h)}{\partial \theta }\right]$$

where E is the expectation.

Unsupervised pre-training with a large amount of unlabeled data is introduced for initializing the RBM [18]. To reconstruct ship-radiated noise accurately, the hidden units of RBM must contain information about aspects of the data that are not relevant to its classification. To remove the redundant features, competitive learning and network pruning based on mutual information is introduced to compress the network.

### 2.2. Competitive Restricted Boltzmann Machine

In order to increase the activation level of inactivated hidden units and reduce the number of network parameters, competitive learning [19,20] is used to cluster the hidden units, so that the hidden units in the same group share the similar weights. Shen [15] grouped hidden units of RBM based on mutual information, but they can’t find out the corresponding category of each group and it is inefficient to calculate the mutual information. However, a given hidden unit of well-trained RBM has different activation level on different categories. A category to which a unit is responding maximally could be a good first-order representation of what the unit is doing [21]. These hidden units can be viewed as feature detectors of that category.

A statistical method is adopted by calculating the score of a given hidden unit driven by different categories. RBM with *m* hidden units can be trained by training data with *L* categories, where $h={\left(h_{1},h_{2},\ldots ,h_{m}\right)}^{T}$ represent the hidden units and k∈(1,2,…,L) represents the category number. The activation of hidden unit *j* is $h_{j}\left(v,\theta \right)$, which is a function of both parameter θ and input sample v. The score of hidden unit *j* driven by the $k^{th}$ category is:

(3) $$score\left(h_{jk}\right)=\frac{1}{n_{k}}\sum_{p=1}^{n_{k}}h_{j}\left(v_{k}^{p},\theta \right)-\frac{1}{n_{/k}}\sum_{q=1}^{n_{/k}}h_{j}\left({v_{/k}}^{q},\theta \right)$$

where $v_{k}$ is a sample of the $k^{th}$ category, $n_{k}$ is the number of samples in the $k^{th}$ category, $v_{/k}$ is a sample of other categories and $n_{/k}$ is the number of its samples. The hidden units which have the maximum score driven by the $k^{th}$ category will be included in the $k^{th}$ group. In general, each hidden unit belongs to only one group and each group contains at least one hidden unit. Each group is assigned to one of the predetermined categories.

Lateral connections are added between hidden units in different groups to build the competitive layer. Lateral inhibition between groups is from small negative weights. The gradient algorithm is derived to optimize the weights of CRBM, the loss function is:

(4) $$Loss=\frac{1}{2}\sum_{j=1}^{m}{\left[c_{j}-h_{j}\left(v\right)\right]}^{2}$$

The gradient for each grouped weight is calculated and used to update the weights of RBM. Competitive learning is used to identify the shared weights in each group, so that hidden units in the same group will share the similar weights. Weight sharing is determined by integrating competitive learning into the RBM learning algorithm, so that the shared weights can be optimized dynamically according to the loss function during the training procedure.

### 2.3. Network Pruning Based on Mutual Information

**Figure 2:**
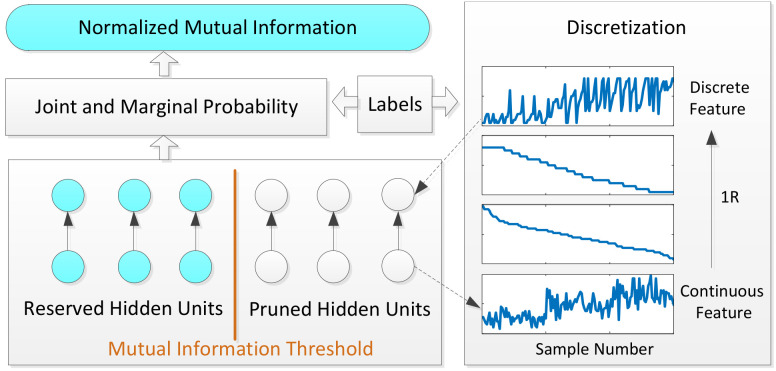
Network pruning based on mutual information. Left part of the diagram represents estimating normalized mutual information (NMI) from learned features. Right part of the diagram is 1R discretization preprocessing step.

A well-trained RBM still contains lots of hidden units that are not relevant to the classification and this will inevitably have negative effects on the recognition accuracy. According to the grouping strategy above, the number of hidden units in different groups may be unbalanced. In order to further improve the classification accuracy together with reducing the network parameters, network pruning based on mutual information is proposed. Figure 2 is the diagram of network pruning based on mutual information.

The network is compressed by pruning the redundant hidden units, and then the informative hidden units are reserved. The connection weights are pruned together with the removed hidden units. The significance of each hidden unit is evaluated by calculating normalized mutual information (NMI) [22,23] with labels. Let *F* denote a feature and *L* represent the labels. NMI(F,L) is defined as:

(5) $$NMI\left(F,L\right)=\frac{H(F)-H(F|L)}{max[H(F),H(L)]}$$

where H(·) is entropy. Entropy of a feature *F* with a probability mass function p(f) is:

(6) $$H\left(F\right)=-\sum_{f}p\left(f\right)\log_{2}p\left(f\right)$$

The conditional entropy H(F|L) is the entropy of a feature *F* conditional on the knowledge of labels *L*. If (F,L)∼p(f,l), H(F|L) is:

(7) $$H\left(F|L\right)=-\sum_{l}\sum_{f}p\left(l,f\right)\log_{2}p\left(f|l\right)$$

In order to estimate entropy of feature *F*, 1R discretization method [24] is used as a preprocessing step. After sorting the continuous values, 1R divides the range of continuous values into a number of disjoint intervals and adjusts the boundaries based on class labels. Each interval should contain a minimum of six instances. For discrete feature variables, both joint and marginal probability tables can be estimated by tallying the samples of categorical variables in the data [25].

It is obvious that NMI(F,L) ranges from 0 to 1. NMI=1 when the two variables are identical and NMI=0 when the two variables are independent. The pruning is deployed group by group to balance the number of hidden units in different groups. All hidden units with NMI below a threshold are removed from the network. The network is retrained to learn the final connection weights for the remaining hidden units. The pruning based on mutual information compresses the competitive network by reducing the number of hidden units and weight vectors.

### 2.4. Compressed Deep Competitive Network

Compressed DCN is constructed by fitting a stack of compressed CRBM and the output of previous layer is input of next layer. Each layer of the network is a CRBM integrated with competitive learning and network pruning. By applying greedy layer-wise training, a compressed DCN with many layers is obtained to represent more complex statistical structure in ship radiated noise. The whole model is then discriminatively fine-tuned by a back propagation algorithm with the target of class labels to further improve classification accuracy [26].

## 3. Experiments and Discussion

### 3.1. Experimental Datasets

**Figure 3:**
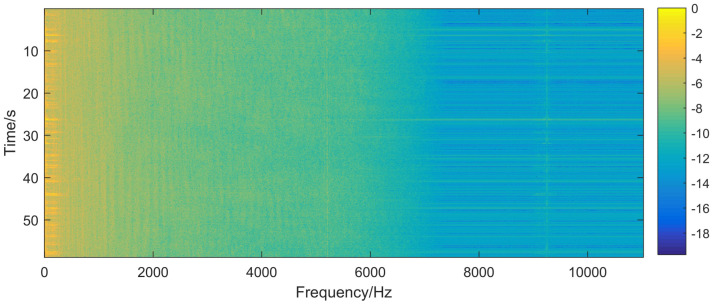
Spectrogram of ship radiated noise.

A dataset of ship radiated noise recorded in the South China Sea was used to verify the proposed model. The dataset can be divided into two categories, including surface targets and autonomous underwater vehicles. The data was acquired using an omnidirectional hydrophone placed at the bottom of a shallow water channel of 30 m depth. The targets were approximately 3.5 km away from the hydrophone, and they moved around the same route with different speeds. Signal to noise ratio (SNR) is 2–3dB. Signals were divided into short frames of 186 ms (4096 samples with the sampling rate of 22,050 Hz). Discrete Fourier Transforms (DFT) of these frames was calculated and only absolute values of the DFT were kept. The final dimension of each sample was 2048 for the symmetry of DFT. Thus, there were 21,530 unlabeled samples and 4210 labeled samples. In the labeled samples, 2100 samples were surface targets and 2110 samples were underwater targets. Labeled dataset was split into two subsets: 50% for training and 50% for testing. Min-max normalization was applied to fit the input of the network. Figure 3 is the spectrogram (logarithmic amplitude) of the input data. The horizontal axis represents the discrete frequency steps, the vertical axis of the spectrogram typically represents time, and the amount of power is represented as the intensity at each time-frequency point [7].

### 3.2. Experimental Procedure

**Figure 4:**
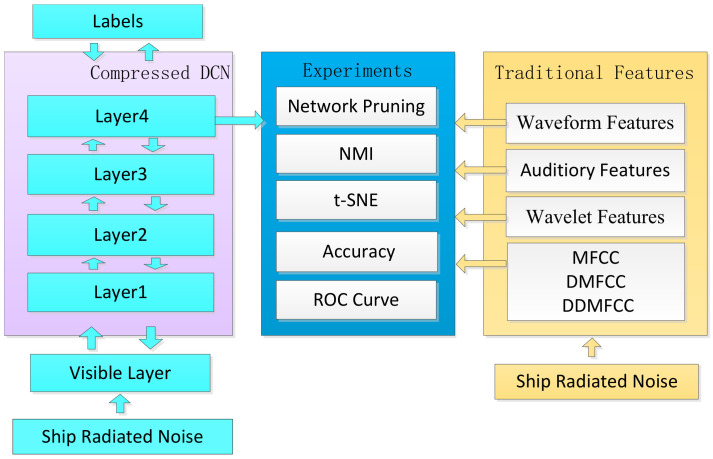
Experiment Procedure.

Training algorithms for deep learning models are iterative and thus require initial point from which to begin the iterations. Normalized initialization was used in our experiments just as Glorot [27] suggested. The initialization method is to initialize the weights of RBM with *n* inputs and *m* outputs by sampling each weight from:

(8) $$W_{ij}∼U\left(-\sqrt{\frac{6}{m+n}},\sqrt{\frac{6}{m+n}}\right)$$

where *U* represents uniform distribution. We used a momentum of 0.5 and mini-batches of 50 randomly selected samples. Grouping strategy, competitive learning and mutual information network pruning were added to Deep-Learning-Toolbox [28] to perform the experiments. The experimental procedure is illustrated in Figure 4.

- Pre-training RBM with 21,530 unlabeled samples with learning rate 0.01.
- Competitive learning with 4210 labeled training data with learning rate 0.001.
- Network pruning with the threshold of average NMI in each layer.
- Greedy layer-wise training and supervised fine-tuning with learning rate 0.01. The initial structure of the DCN is 2048-500-500-50-50. After network pruning, the compressed DCN with structure 2048-163-158-34-31 was obtained.
- The performances of obtained deep features and four widely-used traditional features were compared via t-SNE (t-distributed stochastic neighbor embedding) [29], NMI, classification accuracy and receivers operating characteristic (ROC) curve. In order to make a fair comparison, we used the same classifier for different feature sets. LIB-SVM [30] was used to deal with the small sample size classification problem.

The four traditional feature sets were MFCC features [2], waveform features [4,5], auditory features [31] and wavelet features [6]. MFCC features were extracted by taking the coefficients that make up a Mel-frequency cepstrum. First-order differential Mel-frequency cepstrum coefficients (DMFCC) and second-order differential Mel-frequency cepstrum coefficients (DDMFCC) were calculated. Waveform features were extracted via signal statistical characteristics of zero-crossing wavelength and peek-to-peek amplitude. Auditory features were extracted according to frequency division and masking properties of human auditory system. Wavelet features contained information of entropy of zero-crossing wavelength distribution density of all levels of wavelet signals and low frequency envelope of wavelet decomposition.

### 3.3. Network Pruning Experiments

**Figure 5:**
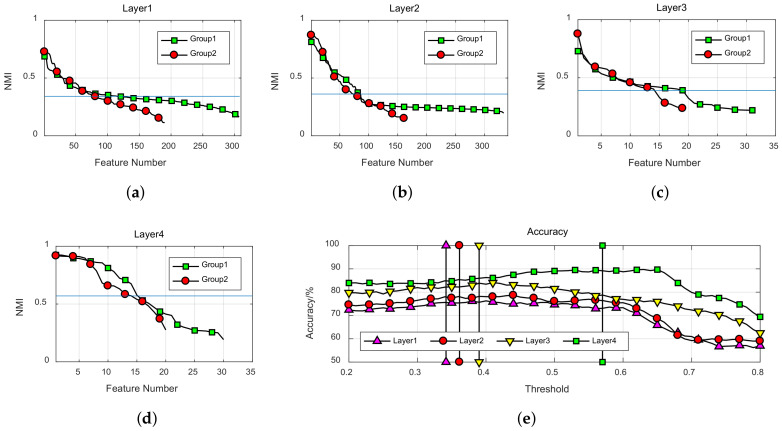
Feature size versus threshold value in each layer, the horizontal lines are the thresholds selected in each layer. (**a**) Layer1; (**b**) Layer2; (**c**) Layer3; (**d**) Layer4; (**e**) Accuracy versus the threshold.

To illustrate relationship between the pruning threshold and feature dimension, NMI of features in each group was arranged in descending order. Feature size versus threshold of each layer is shown in Figure 5 . Horizontal line in each figure is average NMI, which can be used to remove the uninformative features effectively. The numbers of hidden units in different groups are more balanced after network pruning with this threshold.

In order to discuss the influence of the pruning threshold on classification accuracy, classification accuracy of SVM under each pruning threshold was calculated, results are shown in Figure 5e. The vertical lines represent the pruning threshold of each layer. Classification accuracy increases with the increase of threshold at the beginning, because irrelevant features are removed. However, the accuracy begins to decrease when the threshold is too high. Average NMI was used as the threshold in the following experiments.

### 3.4. Clustering Experiments

**Figure 6:**
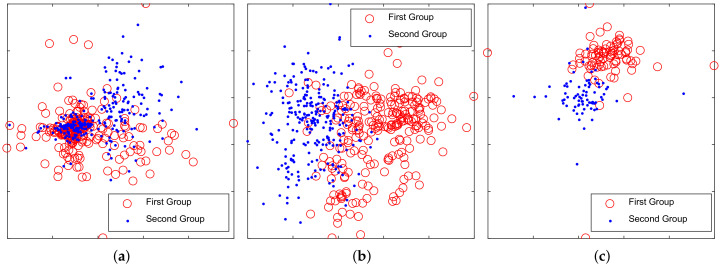
Distribution of weights vectors viewed by t-SNE. (**a**) restricted Boltzmann machine (RBM); (**b**) competitive restricted Boltzmann machine (CRBM); (**c**) The proposed compressed CRBM.

The goal of clustering experiments was to test whether the competitive learning and network pruning can improve discriminative performance. t-SNE [29] feature visualization method was used to observe the distribution of weight vectors in RBM, CRBM and compressed CRBM. The perplexity of the Gaussian kernel in t-SNE is 30. Hidden units of layer1 were divided into two groups according to categories. The scatter diagram of the grouped weight vectors of RBM viewed by t-SNE is shown in Figure 6a. The similar results of CRBM and compressed CRBM are shown in Figure 6b,c, respectively. Both of them are more distributed than that in Figure 6a. The results indicate that CRBM can learn the differences of categories. The compressed CRBM could represent the ship radiated noise by using fewer features than RBM and CRBM.

**Figure 7:**
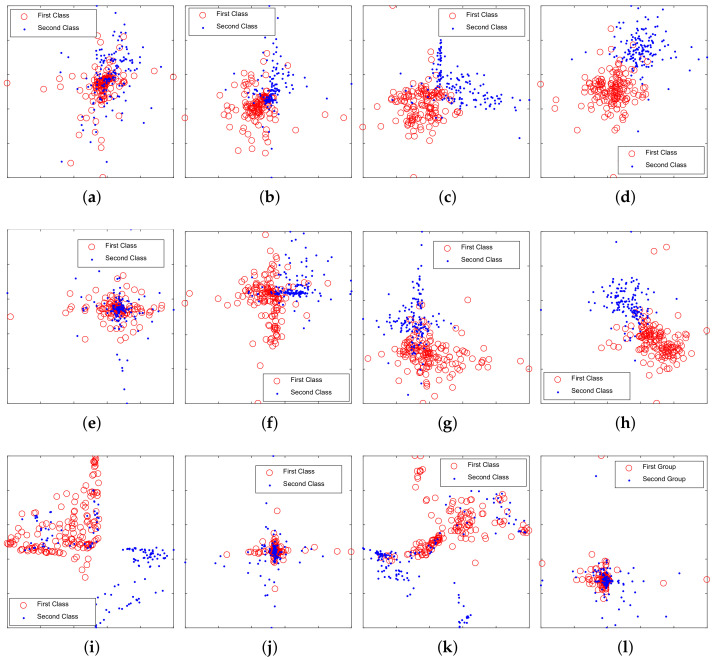
Training samples viewed by t-SNE. (**a**) Layer1 in deep competitive network (DCN); (**b**) Layer2 in DCN; (**c**) Layer3 in DCN; (**d**) Layer4 in DCN; (**e**) pruned Layer1 in the proposed model; (**f**) pruned Layer2 in the proposed model; (**g**) pruned Layer3 in the proposed model; (**h**) pruned Layer4 in the proposed model; (**i**) Mel-frequency cepstral coefficients (MFCC), differential MFCC (DMFCC) and second-order differential MFCC (DDMFCC); (**j**) waveform features; (**k**) auditory features; (**l**) wavelet features.

The distributions of samples described by DCN features, compressed DCN features and traditional features were observed by t-SNE. In total, 150 samples of each category were selected randomly to draw the scatter diagram. Figure 7 shows the comparison of these features. It is obvious that both DCN features and compressed DCN features produce a better distribution than traditional features. The compressed DCN could get a similar result to DCN with fewer features.

### 3.5. Features Evaluation

**Figure 8:**
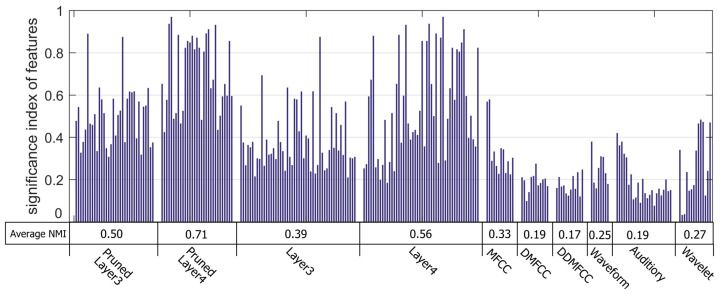
Comparison of each feature via normalized mutual information.

NMI of DCN features, compressed DCN features and traditional features were compared. NMI of each feature and average NMI of each feature set are shown in Figure 8. Features learned by DCN and compressed DCN have higher NMI than traditional features. The average NMI of pruned Layer4 is 0.71, which outperforms other features. There are plenty of DCN features with low NMI, which will have negative effect on the recognition accuracy. Compressed DCN can reduce the feature dimension significantly while reserving the informative features.

### 3.6. Classification Experiments

Classification performances of deep feed-forward neural network (NN), DBN, DCN and Compressed DCN with the same structure were compared. NN was trained with labeled data. DBN, DCN and compressed DCN were pre-trained in an unsupervised phase, followed by supervised fine-tuning. The accuracy and test time are shown in Table 1. Compared with NN, DBN with unsupervised pre-training can significantly improve the classification accuracy. Compared with DBN, competitive learning mechanism in DCN can help improve the classification accuracy. With the help of network pruning, compressed DCN achieved the highest accuracy, which is significantly higher than other methods. Moreover, the test time of compressed DCN is reduced due to the network pruning.

Classification results of DCN features, compressed DCN features and traditional features were compared. SVM classifiers were used to classify the two targets. Parameters of the classifiers were selected by using 10-fold cross validation, in which SVM was trained and tested 10 times repeatedly, with each of the subsamples(randomly partitioned into 10 equal sized) used exactly once as the validation data. Due to the randomness of the initial point of network parameters illustrated in Equation (8), well-trained networks can have varying classification accuracy in different trials depending on the choice of initialization. In order to verify the robustness of the network, average classification accuracy over 10 random trials was conducted to obtain a more stable and repeatable experimental results.

**Figure 9:**
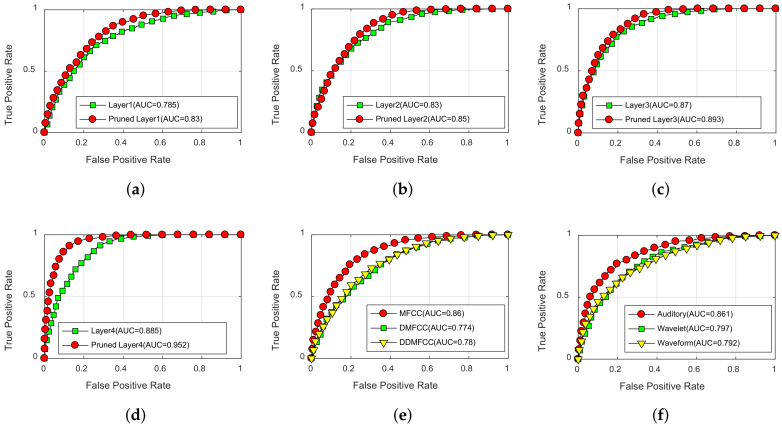
Receivers operating characteristic (ROC) curves of each feature set. (**a**) Layer1 in the proposed model; (**b**) Layer2 in the proposed model; (**c**) Layer3 in the proposed model; (**d**) Layer4 in the proposed model; (**e**) MFCC, DMFCC, DDMFCC; (**f**) Auditory, Waveform, Wavelet.

Assuming that the first class was positive and the second class was negative. ROC curves were constructed by SVM decision function scores obtained on testing data. Figure 9 shows the comparison of ROC curves obtained from DCN features, compressed DCN features and traditional features. The performances of DCN features and compressed DCN features are significantly better than traditional features. Compared with DCN, the compressed DCN has a better performance with fewer features, which indicate that the network pruning can improve the classification accuracy. As shown in Figure 9d, pruned Layer4 can achieve the highest normalized area under ROC curve (AUC). The performance of pruned Layer3 is the second best shown in Figure 9c.

Classification results of SVM are shown in Table 2. For DCN and compressed DCN, the classification accuracy gradually improved as the number of layers increased. The classification accuracy obtained from pruned Layer4 is 89.1%, which is the highest accuracy in our experiments. The features of pruned Layer3 give an accuracy of 83.0%, which perform the second best. Due to the network pruning, the test time can be effectively reduced with the decrease of the feature dimension and this provides conditions for real-time targets classification by sonar devices. Compared with results in Table 1, SVM classifier can achieve equivalent or even higher accuracy on the dataset.

**Figure 10:**
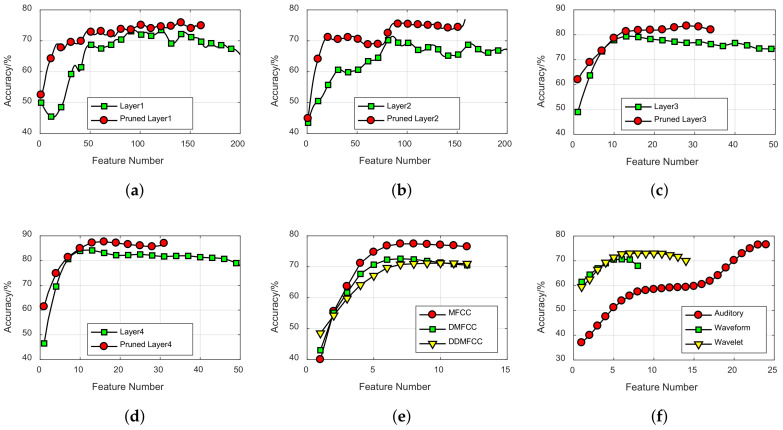
Feature selection results. (**a**) Layer1 in the proposed model; (**b**) Layer2 in the proposed model; (**c**) Layer3 in the proposed model; (**d**) Layer4 in the proposed model; (**e**) MFCC, DMFCC, DDMFCC, (**f**) Auditory, Waveform, Wavelet.

Feature selection algorithm based on NMI was used for each feature set. Features in training dataset were sorted in descending order, and then they were selected incrementally one by one to make up the input feature subsets for SVM classifier, the corresponding features of testing dataset were selected for testing. Results are shown in Figure 10. By applying the feature selection algorithm, the highest accuracy obtained from DCN features is 84.6% shown in Figure 10d and the highest accuracy obtained by traditional features is 78.8% shown in Figure 10e. The pruned layer4 could achieve the highest accuracy of 89.1% which is 5.3% higher than layer4 shown in Figure 10d and 13.1% higher than MFCC shown in Figure 10e. Accuracy obtained from DCN features begins to drop significantly when the feature dimension is too large. However, the accuracy obtained from compressed DCN features increases almost monotonously with the growth of feature dimension, which indicate that the compressed network has fewer redundant features. Compared with DCN, the compressed DCN can reduce feature dimension, while improving the classification accuracy.

## 4. Conclusions

A compressed deep competitive network is presented by integrating competitive learning into the restricted Boltzmann machine learning algorithm and pruning the network based on mutual information. Conclusions are summarized as follows: By applying our algorithm to the underwater acoustic targets recognition, compared with traditional features, the deep competitive network features are more relevant with labels. The deep competitive network can be greatly compressed by pruning redundant hidden units based on mutual information. Support vector machine trained on the features learned by the proposed model can achieve higher classification accuracy with fewer features. The high accuracy and efficiency facilitate the application of the algorithm to real time underwater acoustic targets recognition.

## Figures and Tables

**Table 1:** Classification results of the Neural Network.

Methods	Accuracy/%	Test Time/s
NN	75.5	0.1657
DBN	81.5	0.1624
DCN	82.2	0.1632
Compressed DCN	88.6	0.0974

**Table 2:** Classification results of support vector machine (SVM).

Methods	Features	Dimension	Accuracy/%	Variance/$\times 10^{-3}$	Test Time/s
Traditional	MFCC [2]	12	78.7	4.9	0.0139
	DMFCC [2]	12	71.3	5.3	0.0135
	DDMFCC [2]	12	70.8	5.6	0.0135
	Waveform [4,5]	8	72.4	9.1	0.0552
	Auditory [31]	24	78.4	7.2	0.1071
	Wavelet [6]	14	73.8	7.4	0.0614
DCN	Layer1	500	71.2	4.4	3.8241
	Layer2	500	74.6	4.5	3.8250
	Layer3	50	79.4	3.9	0.1667
	Layer4	50	82.4	3.7	0.1672
Compressed DCN	Pruned Layer1	163	75.4	4.6	0.8067
	Pruned Layer2	158	77.8	4.4	0.7213
	Pruned Layer3	34	83.0	3.8	0.1498
	Pruned Layer4	31	89.1	3.4	0.1423
